# Supplementary material for: Size-Dependent Critical Temperature and Anomalous Optical Dispersion in Ferromagnetic CrI3 Nanotubes
Source: Nanomaterials (Basel). 2019 Jan 26;9(2):153. doi: 10.3390/nano9020153 (PMC6410261; doi:10.3390/nano9020153)
Supplement: Supplementary file 1 [file nanomaterials-09-00153-s001.pdf]

Supplementary Information

Size-Dependent Critical Temperature and Anomalous Optical Dispersion in Ferromagnetic CrI_3 Nanotubes

Mohammed Moaied [1] and Jisang Hong [2,*]

[1] Department of Physics, Faculty of Science, Zagazig University, 44519 Zagazig, Egypt; msmoayed@zu.edu.eg
[2] Department of Physics, Pukyong National University, Busan 608-737, Korea
* Correspondence: hongj@pknu.ac.kr

Stability

To check the dynamical stability of CrI_3 SWNT systems, we calculated the molecular dynamics because it is a reliable tool to check whether the virtual structure is stable.

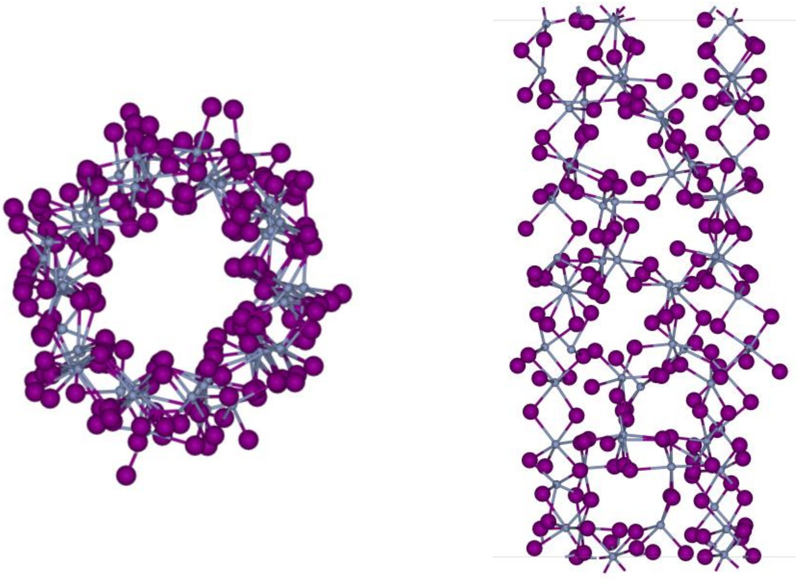

(a)

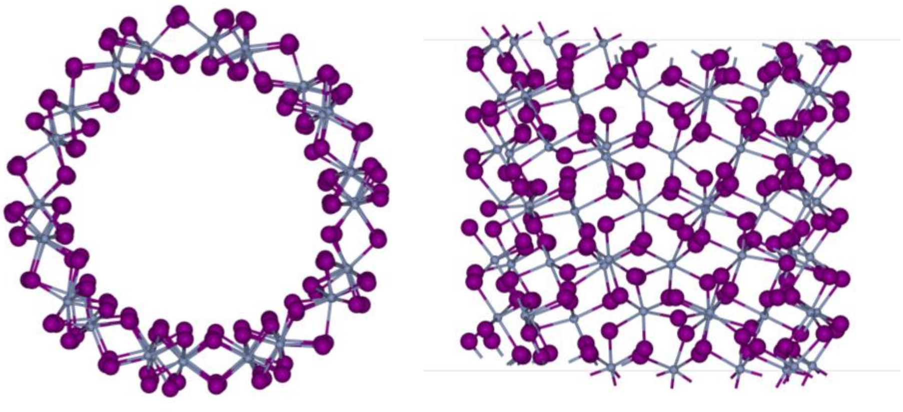

(b)

Figure S1. Snapshots of MD at 5 ps with top and side views at 300 K for (**a**) (5,0)-ZZ NT (**b**) (5,5)-AC NT.

This indicates that these CrI_3 SWNT materials are dynamically stable and can exist as free-standing 1D crystals.

Structural characterization

The increment in the AC tube diameter increases the distortion of the CrI_3 geometry by looking to the pristine 2D CrI_3 layer.

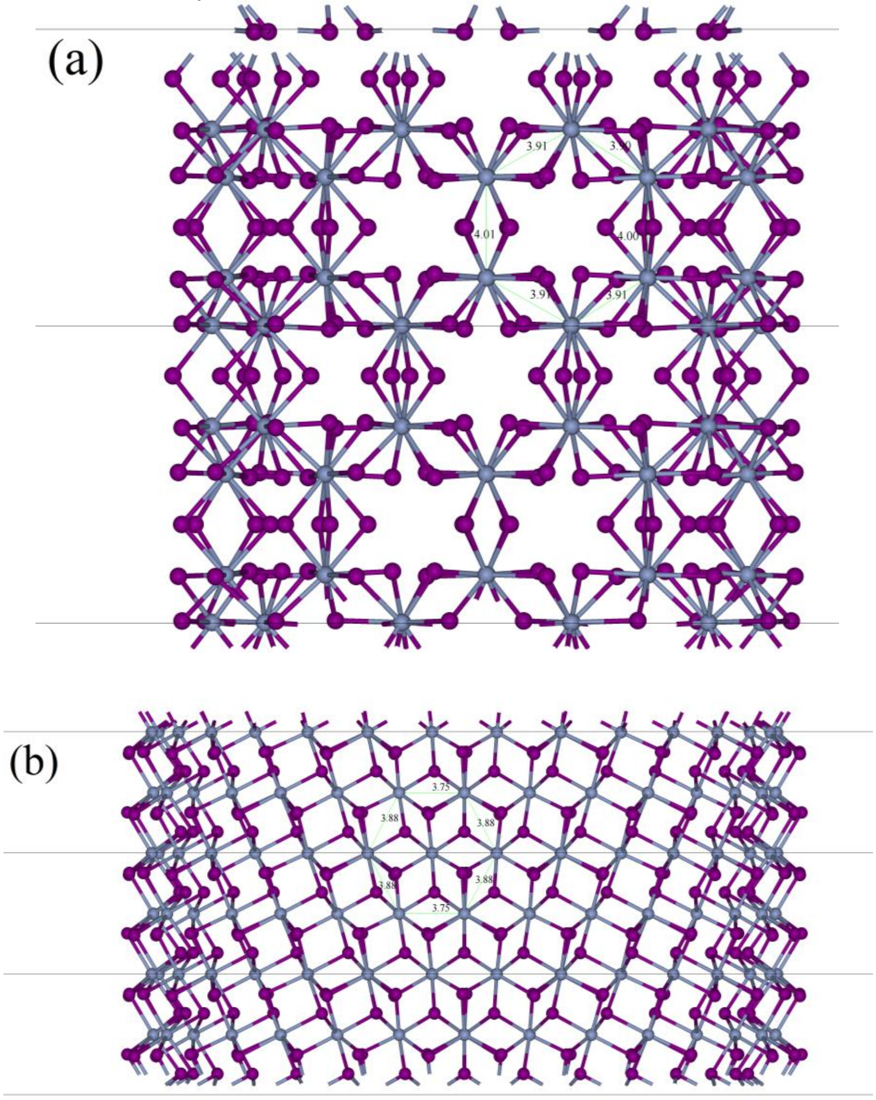

Figure S2. Atomic structure of chromium(III) iodide (CrI_3) NTs: (**a**) illustration of (10,0)-ZZ NT (**b**) (10,10)-AC NT with side views.

Magnetic properties:

We now explore the magnetic ground state. To determine the magnetic ground state, we considered three possible spin configurations; ferromagnetic (FM), nonmagnetic (NM), and antiferromagnetic (AFM) coupling between chromium atoms.

Table S1. Energy difference (in meV) between the FM, NM and AFM for (5,0)-ZZ, (5,5)-AC, (7,0)-ZZ, (10,0)-ZZ, (10,10)-AC, and (12,0)-ZZ CrI_3 SWNT.

Compound	FM (meV)	NM (meV)	AFM (meV)
(5,0)-ZZ	0.00	613	352
(5,5)-AC	0.00	1057	676
(7,0)-ZZ	0.00	1806	1083
(10,0)-ZZ	0.00	3209	1860
(10,10)-AC	0.00	2719	1044
(12,0)-ZZ	0.00	4843	3484

Energy gap

Structural information and energy gap for (7,0)-ZZ NT, and (12,0)-ZZ NT.

Table S2. Structural information of CrI_3 NTs such as lattice parameters (L), the diameter (d), the calculated diameter ($d_{calculated}$), Cr-I bond length (d_{Cr-I}), the interlayer vertical distance between two iodine cylinders (δ), and energy gap (E_g).

Compound	L (Å)	d (Å)	$d_{calculated}$ (Å)	d_{Cr-I} (Å)	δ (Å)	E_g (eV)
(7,0)-ZZ	11.821	16.89	15.22	2.70	2.89	0.702
(12,0)-ZZ	11.821	26.07	26.09	2.68	2.96	0.909

In the zigzag CrI_3 NTs, the band gaps increase from 0.435 eV to 0.9086 eV with increases the diameter. Based on our diameter dependent band gap, we extrapolated the band gap and SI3 shows the calculated result. By extrapolation, we find that the band gap of 2D layer will be about 1.30 eV and this is in good agreement with the previously reported value of 1.2 eV. Indeed, the 2D CrI_3 layer has a band gap of 1.2 eV, and the ZZ NTs would be identical to the monolayer when the tube diameter is infinite.

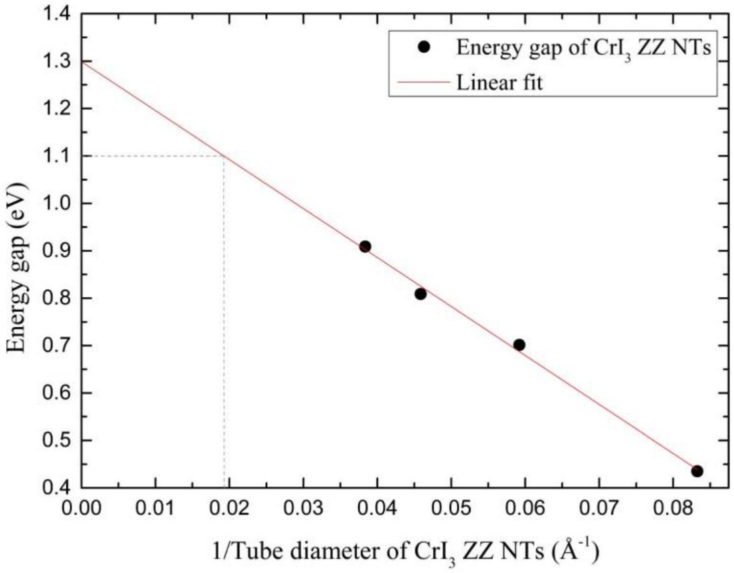

Figure S3. The energy gap of CrI_3 ZZ NTs with respect to diameter reciprocals.

Curie temperature

The magnetic information for CrI_3 (7,0)-ZZ NT, and (12,0)-ZZ NT.

Table S3. Calculated local magnetic moments on the Cr site (M_{Cr}), local magnetic moments on the I site (M_I), total magnetic moments in a unit cell (M_{cell}), exchange energy (E_{ex}), exchange magnetic coupling (J), and Curie temperature (T_c).

Compound	M_{Cr} (μ_B)	M_I (μ_B)	M_{cell} (μ_B)	E_{ex} ($\times 10^{-3}$ eV)	J ($\times 10^{-3}$ eV)	T_c (K)
(7,0)-ZZ	3.37	−0.15	83.19	1082.74	4.30	71
(12,0)-ZZ	3.45	−0.13	143.20	3483.997	8.06	134

The Curie temperature (T_c) is for (7,0)-ZZ NT, and (12,0)-ZZ NT obtained from the temperature dependent magnetization curve.

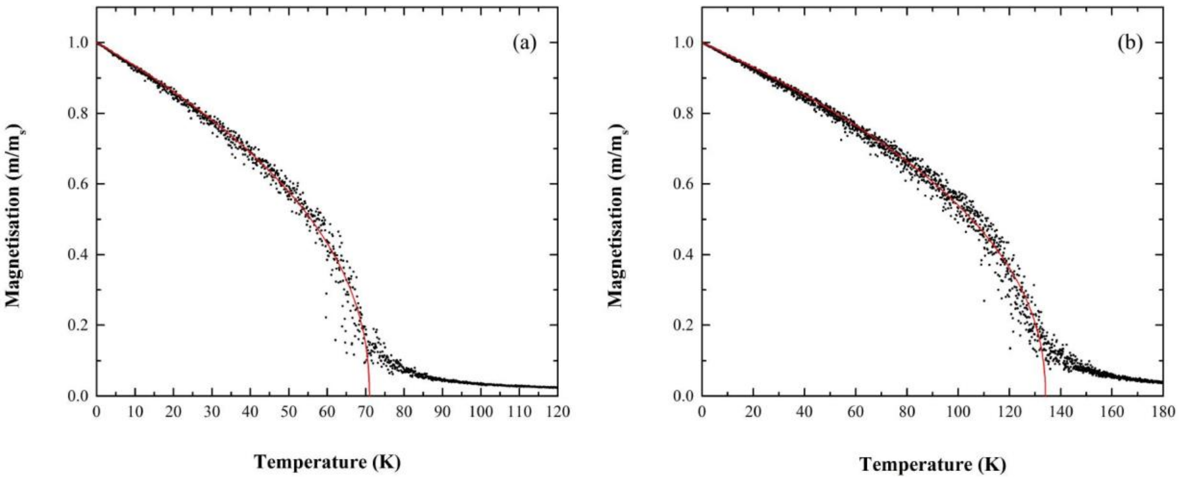

Figure S4. Temperature dependent magnetization curve for CrI_3(a) (7,0)-ZZ NT, and (b) (12,0)-ZZ NT.

From the T_c obtained using Monte Carlo simulation for (5,0)-ZZ NT, (7,0)-ZZ NT, (10,0)-ZZ NT, and (12,0)-ZZ NT, we estimated the T_c by assuming the linear relationship. We can assume that the T_c has a linear relation until the diameter reaches about 5 nm, then our calculation suggests that the room temperature ferromagnetism can be realized in the dimeter about 5 nm ZZ NT and this ZZ NT will have a band gap of 1.1 eV as shows in SI3.
